# Annotated type catalogue of land snails collected from Taiwan (Formosa) in the Natural History Museum, London

**DOI:** 10.3897/zookeys.428.8061

**Published:** 2014-07-23

**Authors:** Chung-Chi Hwang

**Affiliations:** 1 Department of Life Sciences, National University of Kaohsiung, No.700, Kaohsiung University Road, Nan-Tzu District, Kaohsiung 81148, Taiwan

**Keywords:** Type specimen, taxonomy, Taiwan, Gastropoda, land snail

## Abstract

The present catalogue lists the type specimens of land snail species, collected from Taiwan and deposited in the Natural History Museum, London. Thirty-seven nominal species described by Pfeiffer, Adams, Nevill, Moellendorff, Godwin-Austen and Gude were traced. I present here information on type status, collection data obtained from the registers and labels of each collection, and annotations on the current taxonomic affiliation. Lectotypes of 28 nominal (sub)species were newly designated. One holotype was fixed originally and two holotypes newly fixed by monotypy. Syntypes of two species and paralectotypes of three species were also discovered in the Museum. No specimen of the species *Pupina adamsi* Sowerby, 1878, which was supposed to be deposited in the NHM, was found. Pictures of the name-bearing types are provided for further research on biodiversity of the island.

## Introduction

Taxonomic research on historic collections is essential for the modern study of taxonomy and biodiversity. Current taxonomic studies using morphological or molecular methods, e.g., Barcode of Life (Hebert et al. 2003), are among the important issues of organismal, as well as ecological and genetic diversity. However, these attempts will not achieve practical results without a correct identification of species. The most common challenge in current studies of land snail biodiversity in Taiwan is that the sampled specimens cannot be identified correctly and confidently, especially within microsnail taxa such as the diplommatinids. Researchers face similar problems concerning large snail groups such as the clausiliids (Hwang 2005). This situation is mainly due to a lack of type specimens for comparisons since most of the historic types are deposited abroad. The oversimplified descriptions and imprecise illustrations in the original publications from the 19th and early 20th centuries are not practical for modern research. Before complete systematic revisions of each group are possible, concise and updated reports on these types are urgently needed for biodiversity studies in Taiwan. Most of the types collected from Taiwan and deposited in the Naturmuseum Senckenberg, Frankfurt am Main have been reported and photographed in a series of publications by Zilch (e.g., 1953, 1966a, 1966b, 1968). Types described in the 1940s and deposited in the Nishinomiya Shell Museum, Japan, have been catalogued and photographed by Habe and Inaba (1996), Ohara and Otani (2002) and Hwang et al. (2008). Determinations of Taiwanese holotypes and lectotypes deposited in the Academy of Natural Sciences of Philadelphia, U.S.A., were accomplished by Baker (1963, 1964).

The Natural History Museum of London contains many specimens collected in Taiwan (Formosa). A major portion of these are housed in the type series which were previously in the collection of H. Cuming and H. Adams (Adams 1866; Pfeiffer 1866; Gray 1868). Most of these collections from the island were assembled by R. Swinhoe, a British consul and a pioneer of the study of the natural history in Taiwan (Swinhoe 1864, 1865; Fraser 1865, 1866). Pfeiffer (1866) described 13 species of land snails from Cuming’s collection; H. Adams (1866, 1867, 1870, 1872) described 16 species from the collections of the Natural History Museum of London and his own. Some of the types described by other researchers such as Sowerby (1878), Godwin-Austen (1907) and Gude (1907), who worked in or with the NHM, are also deposited in the NHM. These type lots have not been re-examined and catalogued since their original publication. The present report provides the first catalogue and photographs of these type specimens.

## Methods

In preparing this catalogue, type specimens were recognized and verified by comparing information on the specimen labels, the original descriptions, NHM registers and curatorial records of the Mollusca Section, Natural History Museum, London.

The type specimens are listed using modern classifications (Vaught 1989; Bouchet and Rocroi 2005; Hsieh et al. 2013). The collection data obtained from the registers and labels of each collection is provided. The publication dates of those names described in the Proceedings of the Zoological Society of London are corrected according to Duncan (1937). Taxonomic annotations are made when required. Photographs of the types are provided when they have not previously been presented. Type localities are cited as in original descriptions. The modern locality names in Hanyu Pinyin Romanisation are provided in brackets. Dimensions of shells are given as: shell height × shell width. An updated and detailed description of these type materials will be presented elsewhere in systematic studies of their respective groups.

### Institutional abbreviations

NHM, Natural History Museum, London, U.K. (NHM registered specimens are cited as NHMUK); ANSP, Academy of Natural Sciences of Philadelphia, U.S.A.; SMF, Naturmuseum Senckenberg, Frankfurt am Main, Germany.

## Results

I was able to find type specimens of 37 species in the NHM, including all 13 species identified by Pfeiffer (1866) and all 16 by Adams (1866, 1867, 1870, 1872). These specimens are listed in a current taxonomic assignment of nine families including the Cyclophoridae, Diplommatinidae, Pupinidae, Clausiliidae, Streptaxidae, Trochomorphidae, Ariophantidae, Bradybaenidae and Camaenidae. Specimens of *Pupina adamsi* Sowerby, 1878, which are supposedly deposited in the NHM, were not found. Types of eight additional species described by Nevill (1881, two species), Moellendorff (1884, one species), Godwin-Austen (1907, two species), Gude (1907, one species) and Rolle (1911, two species) from various collections were found. Lectotypes of 28 species were newly designated. Paralectotypes of three species, the lectotype of each having already been designated, were found. A holotype of one species was fixed by original designation and holotypes of two species have been fixed by monotypy in the present catalogue. Syntypes of two species were discovered, but no lectotypes were designated as more suitable material for lectotype designation may be kept in the museum where the respective author worked.

Specimens from the collections of shell dealers B. Schmacker (Shanghai, China) and Y. Hirase (Kyoto, Japan) were also found. These species were described by Schmacker and Boettger (1891), Pilsbry (1905) and Pilsbry and Hirase (1905–1906, 1909). Boettger and Pilsbry were the major contributors for description and publication. Due to a lack of evidence, e.g. original labels, of proof of examination of these specimens by Boettger and Pilsbry, these materials were excluded from the type series until further evidence can be found.

I noted the following features in the collection. (1) All specimens are cased in boxes having an originally handwritten label glued onto the bottom. The register information, if any, was also written on the bottoms of the cases. A concise locality, usually “Formosa” only, with or without a collector, was provided. (2) Some specimen lots are labelled “TYPE” using ink and handwriting different from the original labels. Such conditions are supposed to be determined as types and written by a curator or later researchers rather than by the original authors. (3) Ten species described by Adams (1866, 1867, 1870, 1872) were deposited in two batches in the NHM. One batch consists of the specimens that Swinhoe presented to the NHM in 1866, being subsequently examined and named by Adams. The other batch is Adams’ own collection purchased by the NHM in 1878 after his death. Since all specimens were examined by Adams, both are considered as being from the type series.

## Catalogue

### Family CYCLOPHORIDAE

#### Genus *Cyclotus* Swainson, 1840

##### Cyclotus taivanus

Taxon classificationAnimaliaMesogastropodaCyclophoridae

H. Adams, 1870

http://www.taibif.tw/en/catalogue_of_life/page/4e53-43b4-ed74-0962-a6da-f113-5fbb-174b-namecode-405332

Figure 1A

Cyclotus taivanus H. Adams, 1870: 378–379, pl. 27 figs. 11, 11a.

###### Type locality.

Taiwan, Formosa [Taiwan Fu = Tainan City and northern Kaohsiung City] (Swinhoe).

###### Material examined.

*Lectotype*. Formosa, coll. Swinhoe (NHMUK 1871.1.20.9/1), new designation, 10.1 × 16.5 mm, whorls 4.5.

*Paralectotypes*. Formosa, coll. Swinhoe (NHMUK 1871.1.20.9/2–8, 7 shells). Taiwan, Formosa, from collection of H. Adams (NHMUK 1878.1.28.22, 3 shells).

###### Remarks.

The lot NHMUK 1871.1.20.9 was registered and labelled as “Cyclotus formosensis”, but was later published as *Cyclotus taivanus*. Nine specimens were registered, but only eight were found. The specimen corresponding well in size with the measurement of Adams (1870) is designated as the lectotype for the stabilization of the name (ICZN 1999: Art. 74).

**Figure 1.**
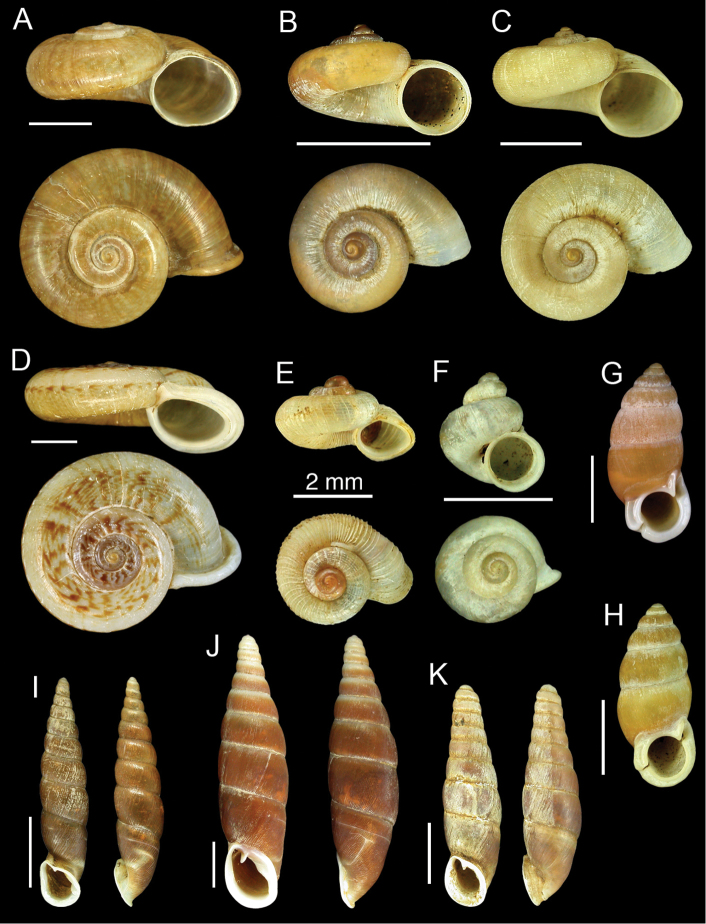
**A** *Cyclotus taivanus* H. Adams, 1870, lectotype NHMUK 1871.1.20.9/1 **B** *Platyrhaphe minutus* (H. Adams, 1866), lectotype NHMUK 1866.5.9.6/1 **C** *Platyrhaphe swinhoei* (H. Adams, 1866), lectotype NHMUK 1866.5.9.7/10 **D** *Ptychopoma wilsoni* (Pfeiffer, 1866), lectotype NHMUK 20040584/1 **E** *Chamalycaeus hungerfordianus* (Nevill, 1881), possible syntype NHMUK 1891.3.17.790 **F** *Dioryx swinhoei* (H. Adams, 1866), lectotype NHMUK 1866.5.9.8/1 **G** *Pupinella swinhoei* (H. Adams, 1866), lectotype NHMUK 1866.5.9.15/1 **H** *Pupinella swinhoei* syn. *Pupinopsis morrisonia* H. Adams, 1872, lectotype NHMUK 1871.1.20.16/1 **I** *Euphaedusa sheridani* (Pfeiffer, 1866), lectotype NHMUK 20040589/1 **J** *Hemiphaedusa exilis* (H. Adams, 1866), lectotype NHMUK 1866.5.9.4/1 **K** *Hemiphaedusa similaris* (H. Adams, 1867), lectotype NHMUK 1878.1.28.10/1. Scale bars = 5 mm, unless otherwise specified above the bar.

#### Genus *Platyrhaphe* Moellendorff, 1890

##### Platyrhaphe minutus

Taxon classificationAnimaliaMesogastropodaCyclophoridae

(H. Adams, 1866)

http://www.taibif.tw/en/catalogue_of_life/page/1451-61f0-132b-71f5-e030-a36b-75a3-1ab7-namecode-315206

Figure 1B

Cyclotus minutus H. Adams, 1866: 318, pl. 33 fig. 10.
Platyrhaphe minutus , Kobelt and Moellendorff 1897: 115.

###### Type locality.

Takow, Formosa [northern Qi-Jin, Kaohsiung City] (Swinhoe)

###### Material examined.

*Lectotype*. Formosa, coll. Swinhoe (NHMUK 1866.5.9.6/1), new designation, 4.4 × 6.9 mm, whorls 4.

*Paralectotypes*. Formosa, coll. Swinhoe (NHMUK 1866.5.9.6/2–3, 2 shells). Formosa, from collection of H. Adams (NHMUK 1878.1.28.73, 3 shells).

###### Remarks.

Adams (1866) described the locality as Takow, Formosa, but the original label has only "Formosa". The specimen corresponding well in size with the measurement of Adams (1866) and having sculptures in good condition is designated as the lectotype for the stabilization of the name (ICZN 1999: Art. 74).

##### Platyrhaphe swinhoei

Taxon classificationAnimaliaMesogastropodaCyclophoridae

(H. Adams, 1866)

http://www.taibif.tw/en/catalogue_of_life/page/4f5b-8cf6-7311-1585-f526-3427-7340-e0db-namecode-315208

Figure 1C

Cyclotus swinhoei H. Adams, 1866: 318, pl. 33 fig. 9.
Platyrhaphe swinhoei , Kobelt and Moellendorff 1897: 116.

###### Type locality.

Takow, Formosa [northern Qi-Jin, Kaohsiung City] (Swinhoe)

###### Material examined.

*Lectotype*. Formosa, coll. Swinhoe (NHMUK 1866.5.9.7/1, new designation, 6.8 × 11.6 mm, whorls 4.5.

*Paralectotype*. Formosa, coll. Swinhoe (NHMUK 1866.5.9.7/2, 1 shell). Formosa, from collection of H. Adams (NHMUK 1878.1.28.231, 1 shells)

###### Remarks.

Adams (1866) described the locality as Takow, Formosa. However, this species was not actually collected there. I think Swinhoe and Adams used Takow to designate the area presently known as Kaohsiung City (Wu et al. 2008; also see remarks for *Oospira formosensis* (Adams, 1866)). The largest specimen corresponds well in size with the measurements of Adams (1866). It is here selected as the lectotype.

#### Genus *Ptychopoma* Moellendorff, 1885

##### Ptychopoma wilsoni

Taxon classificationAnimaliaMesogastropodaCyclophoridae

(Pfeiffer, 1866)

http://www.taibif.tw/en/catalogue_of_life/page/a1f3-b693-4cad-94cc-160c-98ae-38b4-97d0-namecode-405367

Figure 1D

Pterocyclos wilsoni Pfeiffer, 1866: 831, pl. 46 fig. 12. [1865] (Apr. 1866).
Ptychopoma wilsoni , Kobelt and Moellendorff 1897: 88.

###### Type locality.

Formosa [Taiwan] (Swinhoe).

###### Material examined.

*Lectotype*. Formosa, coll. Swinhoe, from collection of H. Cuming (NHMUK 20040584/1). new designation, 9.4 × 22.5 mm, whorls 5.

*Paralectotypes*. Formosa, coll. Swinhoe, from collection of H. Cuming (NHMUK 20040584/2-3, 2 shells).

###### Remarks.

No specimen corresponds in size with the measurements of Pfeiffer (1866). The largest specimen mostly similar to the illustration in Pfeiffer (1866) is designated as the lectotype.

#### Genus *Chamalycaeus* Kobelt & Moellendorff, 1897

##### Chamalycaeus hungerfordianus

Taxon classificationAnimaliaMesogastropodaCyclophoridae

(Nevill, 1881)

http://www.taibif.tw/en/catalogue_of_life/page/a6ad-a451-ead1-6549-84d1-38b3-4b28-fed5-namecode-315080

Figure 1E

Alycaeus hungerfordianus Nevill, 1881: 149–150.
Chamalycaeus hungerfordianus , Kuroda 1941: 81.

###### Type locality.

Tamsui, Formosa [Danshui, New Taipei City] (Hungerford).

###### Material examined.

*Possible syntypes*: Tamsui, Formosa, from collection of Hungerford (NHMUK 1891.3.17.790–791, 2 shells), 2.2–2.5 × 3.6–3.8 mm, whorls 3.75. Also in SMF (Zilch 1957).

###### Remarks.

It is possible that further syntypes may be housed in the Indian Museum, Kolkata (Calcutta), India, where Nevill worked. Due to the lack of definitive evidence that the two specimens in the NHM were actually examined by Nevill, no lectotype designation has been made in the present article.

#### Genus *Dioryx* Benson, 1859

##### Dioryx swinhoei

Taxon classificationAnimaliaMesogastropodaCyclophoridae

(H. Adams, 1866)

http://www.taibif.tw/en/catalogue_of_life/page/e2c6-32a9-1653-e068-3819-bac2-23e3-3913-namecode-315083

Figure 1F

Alycaeus (Dioryx) swinhoei H. Adams, 1866: 318, pl. 33 fig. 11.
Dioryx swinhoei , Kobelt and Moellendorff 1897: 149.

###### Type locality.

Takow, Formosa [northern Qi-Jin, Kaohsiung City] (Swinhoe).

###### Material examined.

*Lectotype*. Formosa, coll. Swinhoe (NHMUK 1866.5.9.8/1), new designation, 6.0 × 5.5 mm, whorls 4.

*Paralectotype*. Formosa, coll. Swinhoe (NHMUK 1866.5.9.8/2, 1 shell).

###### Remarks.

Adams (1866) described the locality as Takow, Formosa, but the original label has only "Formosa". Also see remarks for *Oospira formosensis* (Adams, 1866). The specimen with intact shell is designated as the lectotype.

### Family DIPLOMMATINIDAE

#### Genus *Diplommatina* Benson, 1849

##### Diplommatina hungerfordiana

Taxon classificationAnimaliaMesogastropodaDiplommatinidae

Nevill, 1881

http://www.taibif.tw/en/catalogue_of_life/page/ca4a-ac7e-eeb0-ddb1-72a0-a26f-0975-efa5-namecode-315295

Diplommatina hungerfordiana Nevill, 1881: 150.

###### Type locality.

Kulung, Formosa [should be Keelung, Northern Taiwan] (Hungerford).

###### Material examined.

*Possible syntypes*: Formosa, coll. Hungerford (NHMUK 1891.3.17.724–726, 3 shells), 2.76–3.29 × 1.66–1.76 mm, whorls 5.5–6.5. Also in SMF (Zilch 1953, Figure 186).

###### Remarks.

A photograph of a syntype was provided by Zilch (1953). Also see remarks for *Chamalycaeus hungerfordianus* (Nevill, 1881).

### Family PUPINIDAE

#### Genus *Pupinella* Gray, 1850

##### Pupinella swinhoei

Taxon classificationAnimaliaMesogastropodaPupinidae

H. Adams, 1866

http://www.taibif.tw/en/catalogue_of_life/page/4a7f-dfe1-59ea-ba46-f071-e4d1-7286-f51d-namecode-315419

Figure 1G

Pupinella (Pupinopsis) swinhoei H. Admas, 1866: 318, pl. 32 figs. 12, 12a.

###### Type locality.

Tamsui, Formosa [Danshui, New Taipei City] (Swinhoe).

###### Material examined.

*Lectotype* of *Pupinella swinhoei*. Formosa, coll. Swinhoe (NHMUK 1866.5.9.15/1), new designation, 13 × 5.5 mm, whorls 7. (Figure 1G).

*Paralectotypes* of *Pupinella swinhoei*. Formosa, coll. Swinhoe (NHMUK 1866.5.9.15/2-3, 2 shells). Formosa, from collection of H. Adams (NHMUK 1878.1.28.58, 3 shells)

###### Remarks.

Adams (1866) described the locality of *Platyrhaphe swinhoei* as Tamsui, Formosa, but the original label has only "Formosa". The largest specimen corresponds well in size with the measurements of Adams (1866). It is here selected as the lectotype for the stabilization of the name (ICZN 1999: Art. 74).

##### *Pupinella swinhoei* H. Adams, 1866

###### Syn. Pupinopsis morrisonia

Taxon classificationAnimaliaMesogastropodaPupinidae

H. Adams, 1872

Figure 1H

Pupinopsis morrisonia H. Adams, 1872: 13, pl. 3 fig. 21.

####### Type locality.

Mount Morrison, Formosa [Mt. Yushan] (Swinhoe).

####### Material examined.

*Lectotype* of *Pupinopsis morrisonia*. South Formosa, coll. Swinhoe (NHMUK 1871.1.20.16/1), new designation, 12.5 × 5.3 mm, whorls 6.5. (Figure 1H).

*Paralectotypes* of *Pupinopsis morrisonia*. South Formosa, coll. Swinhoe (NHMUK1871.1.20.16/2, 1 shell). Mt. Morrison, South Formosa, from collection of H. Adams (NHMUK 1878.1.28.50, 3 shells).

####### Remarks.

No specimen corresponds in size with the measurements and illustration of Adams (1872). The specimen similar to the measurements and in the best condition is designated as the lectotype.

##### *Pupinella swinhoei* H. Adams, 1866

###### Syn. Pupina adamsi

Taxon classificationAnimaliaMesogastropodaPupinidae

Sowerby, 1878

Pupina adamsi Sowerby, 1878: sp. 33, pl. 4 fig. 33.

####### Type locality.

Isl. Formosa [Taiwan] (ex. Mus. Brit.)

####### Remarks.

The type specimen of *Pupina adamsi*, which was collected from Formosa and has been stated as being deposited in the NHM (Sowerby 1878), was not found.

### Family CLAUSILIIDAE

#### Genus *Euphaedusa* Boettger, 1877

##### Euphaedusa sheridani

Taxon classificationAnimaliaStylommatophoraClausiliidae

(Pfeiffer, 1866)

http://www.taibif.tw/en/catalogue_of_life/page/c4cf-3a16-c980-f269-6ba0-090e-dccb-2c8d-namecode-316614

Figure 1I

Clausilia sheridani Pfeiffer, 1866: 830–831. [1865] (Apr. 1866).
Euphaedusa sheridani , Yen 1939: 107, pl. 10, fig. 54.

###### Type locality.

Formosa [Taiwan] (Swinhoe).

###### Material examined.

*Lectotype*. Formosa, coll. Swinhoe, from collection of H. Cuming NHMUK 20040589/1), new designation, 16.0 × 3.5 mm, whorls 10.

*Paralectotypes*. Formosa, coll. Swinhoe, from collection of H. Cuming (NHMUK 20040589/2–4, 3 shells; NHMUK 20040589/5, 1 shell, non *sheridani*).

###### Remarks.

Pfeiffer (1866) described a variety with a projecting peristome inside, a narrowed aperture and an inconspicuous inferior lamella. Among the five specimens found, two shells are typical *sheridani* and two shells are of variety. The variations observed in the variety are caused by the thickened callus in the aperture of the fully matured shells. The original description gives a range of shell dimensions.

The largest specimen corresponds well in size with the measurements of Pfeiffer (1866). It is here selected as the lectotype for the stabilization of the name (ICZN 1999: Art. 74). The specimen NHMUK 20040589/5 has similar shell dimensions, but it differs from the other four in having a thin and brownish corneous exterior, a shinier surface, finer striation, a less expanded peristome, a non-protruding aperture and a superior lamella not connected to the spiral lamella. This specimen is closely resembles *Euphaedusa aculus* (Benson, 1842) allies but is not a true *Euphaedusa sheridani*. Because this specimen was examined by Pfeiffer, it should be included in the type series (ICZN 1999: Art. 72.4, 73.2). This specimen is still designated as a paralectotype.

#### Genus *Hemiphaedusa* Boettger, 1877

##### Hemiphaedusa exilis

Taxon classificationAnimaliaStylommatophoraClausiliidae

(H. Adams, 1866)

http://www.taibif.tw/en/catalogue_of_life/page/4ac5-7dd6-1c05-dee6-b6a0-6f0e-a9a7-710b-namecode-316633

Figure 1J

Clausilia (Laciniaria) exilis H. Adams, 1866: 317, pl. 33 fig. 6.
Hemiphaedusa exilis , Kuroda 1941: 140.

###### Type locality.

Tamsui, Formosa [Danshui, New Taipei City] (Swinhoe).

###### Material examined.

*Lectotype*. Formosa, coll. Swinhoe (NHMUK 1866.5.9.4/1), new designation, 27.0 × 7.1 mm, whorls 10.

*Paralectotypes*. Formosa, coll. Swinhoe (NHMUK 1866.5.9.4/2–3, 2 shells). Formosa, from collection of H. Adams (NHMUK 1878.1.28.207, 2 shells).

###### Remarks.

Adams (1866) described the locality as Tamsui, Formosa, but the original label has only "Formosa". The specimen corresponding well in size with the measurements of H. Adams (1866) is designated as the lectotype for the stabilization of the name (ICZN 1999: Art. 74).

##### Hemiphaedusa similaris

Taxon classificationAnimaliaStylommatophoraClausiliidae

(H. Adams, 1867)

http://www.taibif.tw/en/catalogue_of_life/page/8800-40ed-d9b1-5696-861a-c399-1104-4dc5-namecode-316647

Figure 1K

Clausilia (?) *similaris* H. Adams, 1867: 446, pl. 38 fig. 10. [1866] (Apr. 1867).
Hemiphaedusa similaris , Kuroda 1941: 140.

###### Type locality.

Formosa [Taiwan] (Swinhoe).

###### Material examined.

*Lectotype*. Formosa, from collection of H. Adams (NHMUK 1878.1.28.10/1), new designation, 17.3 × 4.8 mm, whorls 10.

*Paralectotypes*. Formosa, from collection of H. Adams (NHMUK 1878.1.28.10/2, 1 shell). Formosa, coll. Swinhoe (NHMUK 1866.5.9.3, 3 shells).

###### Remarks.

None of these type series exactly match with the illustration and measurements in Adams (1867). The specimen in the best condition is here designated as the lectotype.

#### Genus *Oospira* Blanford, 1872

##### Oospira formosensis

Taxon classificationAnimaliaStylommatophoraClausiliidae

(H. Adams, 1866)

http://www.taibif.tw/en/catalogue_of_life/page/6230-62a2-52e0-7159-5ddc-51e0-783b-d62f-namecode-402850

Figure 2A

Clausilia (Phaedusa) formosensis H. Adams, 1866: 317, pl. 33 fig. 7.
Oospira (Formosana) formosensis , Nordsieck 1997: 11.

###### Type locality.

Takow, Formosa [northern Qi-Jin, Kaohsiung City] (Swinhoe)

###### Material examined.

*Lectotype*. Formosa, coll. Swinhoe (NHMUK 1866.5.9.5/1), new designation, 26.1 × 7.0 mm, whorls 10.

*Paralectotypes*. Formosa, coll. Swinhoe (NHMUK 1866.5.9.5/2–3, 2 shells). Formosa, from collection of H. Adams (NHMUK 1878.1.28.245, 1 shell).

###### Remarks.

The specimen corresponding well in size with the measurements of Adams (1866) is designated as the lectotype for the stabilization of the name (ICZN 1999: Art. 74). Adams (1866) described the locality as Takow, Formosa. This species has recently been recorded by Hsieh et al. (2013) in the Liu-gui and Mei-nong areas in eastern Kaohsiung City, which were visited by Swinhoe during his collection trip through southern Taiwan. This species was unlikely to have been collected in Takow (coastal areas of Kaohsiung City in 1866). Apparently, Swinhoe or Adams used Takow to represent an area approximating present-day Kaohsiung City. The same situation was also observed for the type locality of *Platryhaphe swinhoi* and *Dioryx swinhoei*.

**Figure 2.**
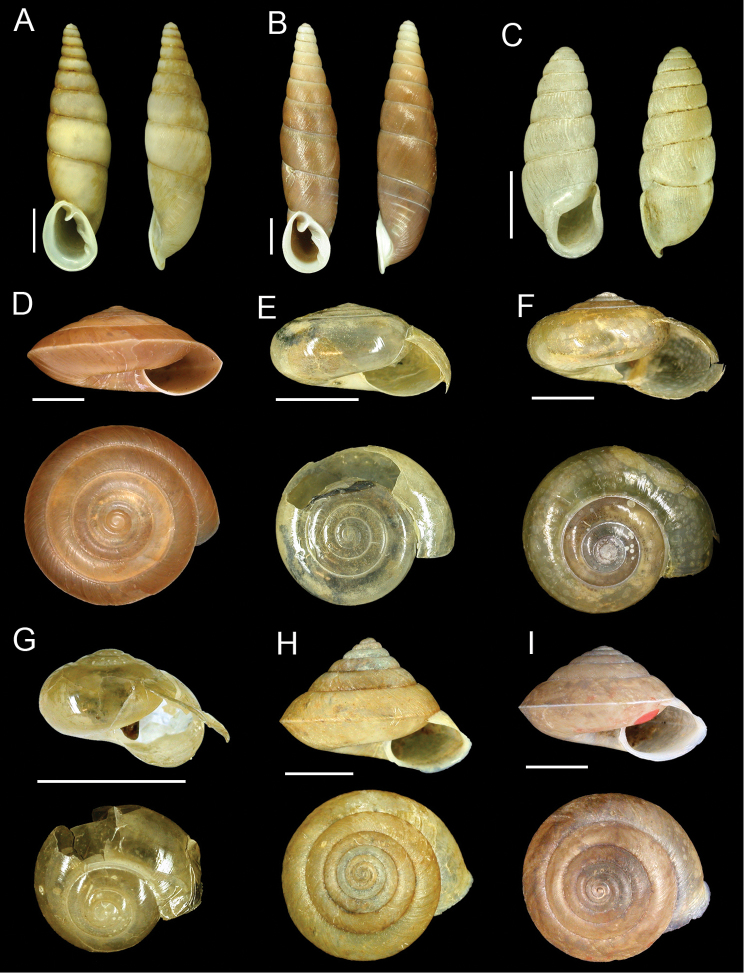
**A** *Oospira formosensis* (H. Adams, 1866), lectotype NHMUK 1866.5.9.5/1 **B** *Oospira swinhoei* (Pfeiffer, 1866), lectotype NHMUK 20040583/1 **C** *Elma swinhoei* (H. Adams, 1866), lectotype NHMUK 1866.5.9.14/1 **D** *Videnoida shermani* (Pfeiffer, 1866), lectotype NHMUK 20040584/1 **E** *Petalochlamys formosanus* (Schmacker & Boettger, 1891) syn. *Petalochlamys hypograpta* Godwin-Austen, 1907, holotype NHMUK 1903.7.1.1713 **F** *Petalochlamys vesta* (Pfeiffer, 1866), lectotype NHMUK 20040580 **G** *Ovachlamys fulgens* (Gude, 1900) syn. *Lamprocystis fulgida* Godwin-Austen, 1907, holotype NHMUK 1903.7.1.1714 **H** *Aegista fulvicans* (H. Adams, 1866), lectotype NHMUK 1866.5.9.10/1 **I** *Aegista granti* (Pfeiffer, 1866), lectotype NHMUK 20040579. Scale bars = 5 mm.

##### Oospira swinhoei

Taxon classificationAnimaliaStylommatophoraClausiliidae

(Pfeiffer, 1866)

http://www.taibif.tw/en/catalogue_of_life/page/f228-43fd-a0a7-32f2-aaea-1ba6-e70c-854d-namecode-402851

Figure 2B

Clausilia swinhoei Pfeiffer, 1866: 830, pl. 46 fig. 11. [1865] (Apr. 1866).
Oospira (Formosana) swinhoei , Nordsieck 1997: 11.

###### Type locality.

Formosa [Taiwan] (Swinhoe)

###### Material examined.

*Lectotype*. Formosa, coll. Swinhoe, from collection of H. Cuming (NHMUK 20040583/1), new designation, 35.0 × 8.1 mm, whorls 10.

*Paralectotypes*. Formosa, coll. Swinhoe, from collection of H. Cuming (NHMUK 20040583/2–3, 2 shells).

###### Remarks.

The original description gives a range of shell dimensions. The largest specimen is designated as the lectotype.

### Family STREPTAXIDAE

#### Genus *Elma* H. Adams, 1866

##### Elma swinhoei

Taxon classificationAnimaliaStylommatophoraStreptaxidae

(H. Adams, 1866)

http://www.taibif.tw/en/catalogue_of_life/page/45f2-e28e-c620-b0cb-2306-5af5-f514-0395-namecode-402881

Figure 2C

Ennea (Elma) swinhoei H. Adams, 1866: 317–318, pl. 33 fig. 8.
Elma swinhoei , Kuroda 1941: 148.

###### Type locality.

Tamsui, Formosa [Danshui, New Taipei City] (Swinhoe)

###### Material examined.

*Lectotype*. Formosa, coll. Swinhoe (NHMUK 1866.5.9.14/1), new designation, 15.7 × 6.1 mm, whorls 9.

*Paralectotypes*. Formosa, coll. Swinhoe (NHMUK 1866.5.9.14/2–3, 2 shells). Tamsui, Formosa, coll. Swinhoe, from collection of H. Adams (NHMUK 1878.1.28.30, 3 shells).

###### Remarks.

The largest specimen corresponds well in size with the measurements of Adams (1866). It is here selected as the lectotype for the stabilization of the name (ICZN 1999: Art. 74).

### Family TROCHOMORPHIDAE

#### Genus *Videnoida* Habe, 1955

##### Videnoida shermani

Taxon classificationAnimaliaStylommatophoraTrochomorphidae

(Pfeiffer, 1866)

http://www.taibif.tw/en/catalogue_of_life/page/af4c-5a14-6587-4495-c500-e4ba-0b5f-8a08-namecode-316729

Figure 2D

Helix shermani Pfeiffer, 1866: 828, pl. 46 fig. 5. [1865] (Apr. 1866).
Videnoida shermani , Chang 1994: 17.

###### Type locality.

Formosa [Taiwan] (Swinhoe).

###### Material examined.

*Lectotype*. Formosa, coll. Swinhoe, from collection of H. Cuming (NHMUK 20040584/1), new designation, 8.7 × 19.3 mm, whorls 7.

*Paralectotypes*. Formosa, coll. Swinhoe, from collection of H. Cuming (NHMUK 20040584/2–3, 2 shells).

###### Remarks.

The largest specimen mostly similar to the illustration in Pfeiffer (1866) is designated as the lectotype.

### Family ARIOPHANTIDAE

#### Genus *Petalochlamys* Godwin-Austen, 1907

##### *Petalochlamys formosanus* (Schmacker & Boettger, 1891)

###### Syn. Petalochlamys hypograpta

Taxon classificationAnimaliaStylommatophoraAriophantidae

Godwin-Austen, 1907

http://www.taibif.tw/en/catalogue_of_life/page/7b11-e296-0f06-7c00-8eef-6282-8085-ec60-namecode-316691

Figure 2E

Macrochlamys (Petalochlamys) formosana var. *hypograpta* Godwin-Austen, 1907: 206, 212–214, pl. 115 figs. 2, 2b, pl. 116 figs. 5, 5b. (Apr. 1907).

####### Type locality.

South Formosa [southern Taiwan] (Hirase)

####### Material examined.

*Holotype*. Southern Formosa, from collection of Hirase (NHMUK 1903.7.1.1713), designated by monotypy, 11.0 × 6.3 mm, whorls 5.5. (Figure 2E).

####### Remarks.

This name was first recommended by Pilsbry for specimens from Hirase’s collection (Hirase 1908). However, Godwin-Austen (1907) received a specimen from Hirase, reported on its reproductive system and assigned it as the type species of the genus *Petalochlamys*. Hirase (1908: 16, published on 20 Jan. 1908) described his own specimens as a new variety of the same name in Japanese, on the basis of specimens collected from Hotawa; however, he also cited Godwin-Austen’s study in a Japanese translation. Therefore, Godwin-Austen’s 1907 publication unintentionally took precedence over Hirase’s work, thereby claiming the authorship of this name. Being the type species of the genus, the name was thus elevated to the rank of species (ICZN 1999: Art. 61.4).

###### Petalochlamys vesta

Taxon classificationAnimaliaStylommatophoraAriophantidae

(Pfeiffer, 1866)

http://www.taibif.tw/en/catalogue_of_life/page/206a-098e-58ca-00df-95c8-1038-5142-b787-namecode-316694

Figure 2F

Helix vesta Pfeiffer, 1866: 828, pl. 46 fig. 9. [1865] (Apr. 1866).
Petalochlamys vesta , Matsuda 1924: 49.

####### Type locality.

Formosa [Taiwan] (Swinhoe)

####### Material examined.

*Lectotype*. Formosa, coll. Swinhoe, from collection of H. Cuming (NHMUK 20040580), new designation, 8.5 × 16 mm, whorls 5.5.

####### Remarks.

Although only one specimen is found in the NHM, it should be designated as lectotype under ICZN (1999: Rec. 73F).

#### Genus *Ovachlamys* Habe, 1946

##### *Ovachlamys fulgens* (Gude, 1900)

###### Syn. Lamprocystis fulgida

Taxon classificationAnimaliaStylommatophoraAriophantidae

Godwin-Austen, 1907

http://www.taibif.tw/en/catalogue_of_life/page/6f48-0a31-58ff-26e8-8637-e924-fbe9-9a3a-namecode-316684

Figure 2G

Macrochlamys fulgens Gude, 1900: 75, pl. 8 figs. 24–26.
Lamprocystis ? *fulgida* Godwin-Austen, 1907: 214, pl. 115 figs. 3–3f, pl. 116 figs. 6–6a.

####### Type locality.

South Formosa [southern Taiwan] (Hirase)

####### Material examined.

*Holotype* of *Lamprocystis fulgida*. South Formosa, from collection of Hirase (NHMUK 1903.7.1.1714), designated by monotypy, 4 × 7.3 mm, whorls 4.5.

####### Remarks.

Godwin-Austen (1907) described the specimen as being 9.75 mm in shell width. By comparing his measurement with mine, I concluded that his was probably an inadvertent error.

### Family BRADYBAENIDAE

#### Genus *Aegista* Albers, 1850

##### Aegista fulvicans

Taxon classificationAnimaliaStylommatophoraBradybaenidae

(H. Adams, 1866)

http://www.taibif.tw/en/catalogue_of_life/page/7c64-c58c-12eb-ac55-a7cd-a096-5667-5d70-namecode-404845

Figure 2H

Helix (Plectotropis) fulvicans H. Adams, 1866: 316, pl. 33 fig. 2.
Aegista (Plectotropis) fulvicans , Kuroda 1941: 147.

###### Type locality.

Tamsui, Formosa [Danshui, New Taipei City] (Swinhoe)

###### Material examined.

*Lectotype*. Formosa, coll. Swinhoe (NHMUK 1866.5.9.10/1), new designation, 9.9 × 14 mm, whorls 7.

*Paralectotypes*. Formosa, coll. Swinhoe (NHMUK 1866.5.9.10/2–3, 2 shells). Formosa, from collection of H. Adams (NHMUK 1878.1.23.204, 2 shells).

###### Remarks.

Adams (1866) described the locality as Tamsui, Formosa, but the original label has only "Formosa". The largest specimen mostly similar to the illustration in Adams (1866) is designated as the lectotype.

##### Aegista granti

Taxon classificationAnimaliaStylommatophoraBradybaenidae

(Pfeiffer, 1866)

http://www.taibif.tw/en/catalogue_of_life/page/7c64-c58c-12eb-ac55-a7cd-a096-5667-5d70-namecode-404845

Figure 2I

Helix granti Pfeiffer, 1866: 828–829, pl. 46 fig. 10. [1865] (Apr. 1866).
Aegista (Plectotropis) granti , Kuroda 1941: 147.

###### Type locality.

Formosa [Taiwan] (Swinhoe)

###### Material examined.

*Lectotype*. Formosa, coll. Swinhoe, from collection of H. Cuming (NHMUK 20040579), new designation, 8.9 × 14.4 mm, whorls 6.5.

###### Remarks.

Although only one specimen is found in the NHM, it should be designated as lectotype under ICZN (1999 Rec. 73F).

#### Genus *Pseudobuliminus* Gredler, 1887

##### Pseudobuliminus incertus

Taxon classificationAnimaliaStylommatophoraBradybaenidae

(Pfeiffer, 1866)

http://www.taibif.tw/en/catalogue_of_life/page/ded5-2c76-8885-2134-814e-8b5e-d842-c8e9-namecode-404771

Figure 3A

Bulimus incertus Pfeiffer, 1866: 830, pl. 46 fig. 1. [1865] (Apr. 1866).
Pseudobuliminus incertus , Schmacker and Boettger 1891: 163.

###### Type locality.

Formosa [Taiwan] (Swinhoe)

###### Material examined.

*Lectotype*. Formosa, coll. Swinhoe, from collection of H. Cuming (NHMUK 20040582/1), new designation, ca. 10 × 4.5 mm, whorls 9.5 (peristome damaged).

*Paralectotypes*. Formosa, coll. Swinhoe, from collection of H. Cuming (NHMUK 20040582/2–4, 3 shells).

###### Remarks.

The largest specimen corresponds well in size with the measurements of Pfeiffer (1866). It is here selected as the lectotype for the stabilization of the name (ICZN 1999: Art. 74).

**Figure 3.**
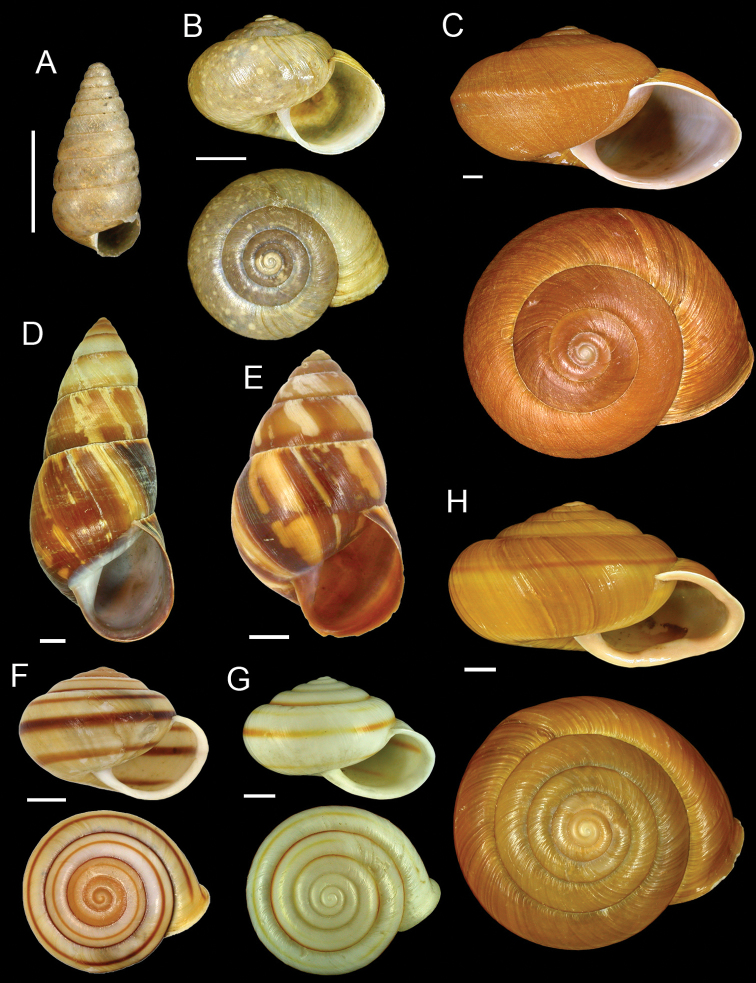
**A** *Pseudobuliminus incertus* (Pfeiffer, 1866), lectotype NHMUK 20040582/1 **B** *Acusta assimilis* (H. Adams, 1866), lectotype NHMUK 1866.5.9.11/1 **C** *Nesiohelix swinhoei* (Pfeiffer, 1866), lectotype NHMUK 20040585/1 **D** *Dolicheulota formosensis* (H. Adams, 1866), lectotype NHMUK 1866.5.9.1/1 **E** *Dolicheulota swinhoei* (Pfeiffer, 1866), lectotype NHMUK 20040586/1 **F** *Satsuma bacca* (Pfeiffer, 1866), lectotype NHMUK 20040577/1 **G** *Satsuma bacca* (Pfeiffer, 1866) syn. *Eulota warburgi* Gude, 1907, holotype NHMUK 1922.8.29.66 **H** *Satsuma bairdi* (H. Adams, 1866), lectotype NHMUK 20040587/1. Scale bars = 5 mm.

#### Genus *Acusta* Albers, 1860

##### Acusta assimilis

Taxon classificationAnimaliaStylommatophoraBradybaenidae

(H. Adams, 1866)

http://www.taibif.tw/en/catalogue_of_life/page/f063-8247-3258-a7f9-1230-23a1-7f12-e537-namecode-316552

Figure 3B

Nanina (Acusta) assimilis H. Adams, 1866: 316, pl. 33 fig. 1.
Acusta assimilis , Chang 1984: 18.

###### Type locality.

Takow, Formosa [northern Qi-Jin, Kaohsiung City] (Swinhoe)

###### Material examined.

*Lectotype*. Formosa, coll. Swinhoe (NHMUK 1866.5.9.11/1), new designation, 13.7 × 19.4 mm, whorls 5.75.

*Paralectotypes*. Formosa, coll. Swinhoe (NHMUK 1866.5.9.11/2–3, 2 shells).

###### Remarks.

Adams (1866) described the locality as Takow, Formosa, but the original label has only "Formosa". The lot NHMUK 1866.5.9.11 was first registered and labelled as “Nanina propinqua”, then later published as *Nanina assimilis*. None of these shells exactly match with the measurements of Adams (1866). The specimen in the best condition is designated as the lectotype for the stabilization of the name (ICZN 1999: Art. 74).

##### Acusta toyenmongaiensis

Taxon classificationAnimaliaStylommatophoraBradybaenidae

Rolle, 1911

Acusta toyenmongaiensis Rolle, 1911: 32.

###### Type locality.

Toyenmongai, Formosa [Dong-yuan-men-jie, Tainan = central area of modern-day Tainan City] (Rolle?)

###### Material examined.

*Lectotype*. Toyenmongai, Formosa (SMF 7405), designated by Zilch (1968, pl. 7, fig. 27).

*Paralectotypes*. Formosa (NHMUK 1912.8.16.131–133, 3 shells, “original specimen”), 11.3–12.3 × 13.3–14.2 mm, whorls 5.25–5.5. Also in SMF.

#### Genus *Nesiohelix* Kuroda & Emura, 1943

##### Nesiohelix swinhoei

Taxon classificationAnimaliaStylommatophoraBradybaenidae

(Pfeiffer, 1866)

http://www.taibif.tw/en/catalogue_of_life/page/6229-bc2d-cbaa-81c2-e0f7-c761-0936-09a5-namecode-316578

Figure 3C

Helix swinhoei Pfeiffer, 1866: 829, pl. 46 fig. 6. [1865] (Apr. 1866).
Nesiohelix swinhoei , Kuroda 1941: 148.

###### Type locality.

Formosa [Taiwan] (Swinhoe).

###### Material examined.

*Lectotype*. Formosa, coll. Swinhoe, from collection of H. Cuming (NHMUK 20040585/1, large form), new designation, 42.7 × 75.3 mm, whorls 5.5.

*Paralectotypes*. Formosa, coll. Swinhoe, from collection of H. Cuming (NHMUK 20040585/2, 1 shell, large form, 50.9 × 68.1 mm, whorls 5.25; NHMUK 20040585/3–4, 2 shells, small form, 32.4–32.5 × 50.8–54.5 mm, whorls 4.75–5.0).

###### Remarks.

The shell sizes of these four specimens do not agree with Pfeiffer’s original measurements of 27–28 × 52–58 mm for the typical form and 25 × 46 mm for the minor form. I consider Pfeiffer’s measurements to be incorrect. The largest specimen is designated as the lectotype for the stabilization of the name (ICZN 1999: Art. 74).

#### Genus *Dolicheulota* Pilsbry, 1901

##### Dolicheulota formosensis

Taxon classificationAnimaliaStylommatophoraBradybaenidae

(H. Adams, 1866)

http://www.taibif.tw/en/catalogue_of_life/page/d4d5-9c75-7cde-9aa3-c80d-6ae0-ec2b-eda7-namecode-316574

Figure 3D

Bulimus (Amphidromus) formosensis H. Adams, 1866: 317, pl. 33 fig. 5.
Dolicheulota formosensis , Pilsbry and Hirase 1906: 735.

###### Type locality.

Tamsui Mountains, Formosa [Danshui, New Taipei City] (Swinhoe).

###### Material examined.

*Lectotype*. Formosa, coll. Swinhoe (NHMUK 1866.5.9.1/1), new designation, 55.4 × 24 mm, whorls 8.

*Paralectotypes*. Formosa, coll. Swinhoe (NHMUK 1866.5.9.1/2–3, 2 shells).

###### Remarks.

Adams (1866) described the locality as Tamsui Mountains, Formosa, but the original label has only "Formosa". The largest specimen corresponding well in size with the measurements of H. Adams (1866) is designated as the lectotype for the stabilization of the name (ICZN 1999: Art. 74).

##### Dolicheulota swinhoei

Taxon classificationAnimaliaStylommatophoraBradybaenidae

(Pfeiffer, 1866)

http://www.taibif.tw/en/catalogue_of_life/page/291f-4ce7-4fad-078f-d5bb-38ba-7785-3f53-namecode-316575

Figure 3E

Bulimus swinhoei Pfeiffer, 1866: 830, pl. 46 figs. 2, 2a. [1865] (Apr. 1866).
Dolicheulota swinhoei Pilsbry & Hirase, 1906: 735.

###### Type locality.

Formosa [Taiwan] (Swinhoe)

###### Material examined.

*Lectotype*. Formosa, coll. Swinhoe, from collection of H. Cuming (NHMUK 20040586/1), new designation, 37.1 × 21.4 mm, whorls 7.

*Paralectotypes*. Formosa, coll. Swinhoe, from collection of H. Cuming (NHMUK 20040586/2–3, 2 shells, one bleached and the other immature).

###### Remarks.

The specimen in the best condition is designated as the lectotype for the stabilization of the name (ICZN 1999: Art. 74).

### Family CAMAENIDAE

#### Genus *Satsuma* A. Adams, 1868

##### Satsuma bacca

Taxon classificationAnimaliaStylommatophoraCamaenidae

(Pfeiffer, 1866)

http://www.taibif.tw/en/catalogue_of_life/page/3b47-78d4-0970-b4b4-ae3c-e171-67c8-d0cc-namecode-316610

Figure 3F

Helix bacca Pfeiffer, 1866: 829, pl. 46 fig. 8. [1865] (Apr. 1866).
Satsuma bacca , Richardson 1985: 268.

###### Type locality.

Formosa [Taiwan] (Swinhoe).

###### Material examined.

*Lectotype* of *Helix bacca*. Formosa, coll. Swinhoe, from collection of H. Cuming (NHMUK 20040577/1), new designation, 16.6 × 24.5 mm, whorls 5.5. (Figure 3F)

*Paralectotype* of *Helix bacca*. Formosa, coll. Swinhoe, from collection of H. Cuming (NHMUK 20040577/2, 1 shell, immature).

###### Remarks.

This species was usually considered as a member of *Pancala* Kuroda & Habe, 1949. Hwang (2011) transferred it to the current genus because of genital and conchological similarities. Besides, *Pancala* is preoccupied by a dipteran genus (Enderlein 1936). The only mature specimen is designated as lectotype.

##### *Satsuma bacca* (Pfeiffer, 1866)

###### Syn. Eulota (Euhadra) warburgi

Taxon classificationAnimaliaStylommatophoraBradybaenidae

Gude, 1907

Figure 3G

Eulota (Euhadra) warburgi Gude, 1907: 164–165, figs. 1, 2.

####### Type locality.

Dunes at Long-Krau, South Formosa [coastal area of Northwest Hengchung Peninsula] (Warburg)

####### Material examined.

*Holotype* of *Eulota (Euhadra) warburgi*. Dunes at Long-Krau, Southern Formosa, coll. Warburg, Feb. 1888, from collection of Naturhistorisches Museum, Hamburg (NHMUK 1922.8.29.66), original designation, 19.2 × 30.5 mm, whorls 5.75. (Figure 3G)

###### Satsuma bairdi

Taxon classificationAnimaliaStylommatophoraCamaenidae

(H. Adams, 1866)

http://www.taibif.tw/en/catalogue_of_life/page/6293-3ac5-ce65-683e-ef8c-6101-7142-a9e1-namecode-402916

Figure 3H

Helix (Camaena) bairdi H. Adams, 1866: 316, pl. 33 fig. 3.
Satsuma bairdi , Richardson 1985: 268.

####### Type locality.

Tamsui, Formosa [Danshui, New Taipei City] (Swinhoe)

####### Material examined.

*Lectotype*. Formosa, coll. Swinhoe, from collection of H. Cuming (NHMUK 20040587/1), new designation, 22.7 × 40.0 mm, whorls 6.25.

*Paralectotypes*. Formosa, coll. Swinhoe, from collection of H. Cuming (NHMUK 20040587/2–3, 2 shells).

####### Remarks.

Adams (1866) described the locality as Tamsui, Formosa, but the original label has only "Formosa". None of these shells exactly match with the measurements of Adams (1866). The specimen in the best condition is designated as the lectotype for the stabilization of the name (ICZN 1999: Art. 74).

###### Satsuma formosensis

Taxon classificationAnimaliaStylommatophoraCamaenidae

(Pfeiffer, 1866)

http://www.taibif.tw/en/catalogue_of_life/page/8f82-28a2-5e55-911c-d11c-a5ce-1537-af29-namecode-402919

Figure 4A

Helix formosensis Pfeiffer, 1866: 829, pl. 46 fig. 7. [1865] (Apr. 1866).
Satsuma formosensis , Richardson 1985: 270.

####### Type locality.

Formosa [Taiwan] (Swinhoe).

####### Material examined.

*Lectotype*. Formosa, coll. Swinhoe, from collection of H. Cuming (NHMUK 20040578), new designation, 17 × 26 mm, whorls 6.

####### Remarks.

Pfeiffer (1866) mentioned that the shell height is 12–13 mm, but only one specimen is found in the NHM. More than one specimen was probably examined by Pfeiffer. Therefore, this is designated as a lectotype instead of a holotype.

**Figure 4.**
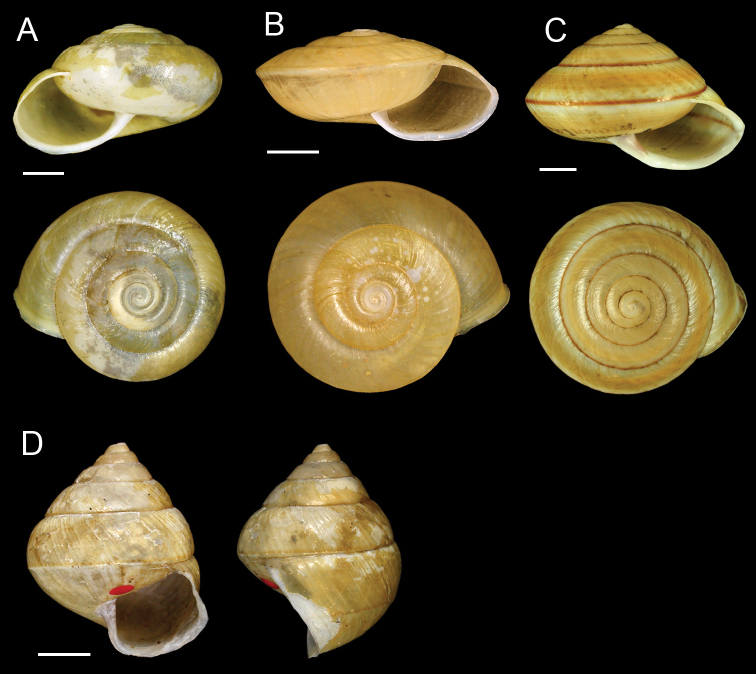
**A** *Satsuma formosensis* (Pfeiffer, 1866), lectotype NHMUK 20040578 **B** *Satsuma mellea* (Pfeiffer, 1866), lectotype NHMUK 20040576/1 **C** *Satsuma succincta* (H. Adams, 1866), lectotype NHMUK 1866.5.9.9/1 **D** *Satsuma sphaeroconus* (Pfeiffer, 1866), lectotype NHMUK 20040575/1. Scale bars = 5 mm.

###### Satsuma mellea

Taxon classificationAnimaliaStylommatophoraCamaenidae

(Pfeiffer, 1866)

http://www.taibif.tw/en/catalogue_of_life/page/8cbf-f9d6-70dd-b1d2-abbf-2f09-2c6e-8166-namecode-402922

Figure 4B

Helix mellea Pfeiffer, 1866: 829–830, pl. 46 fig. 4. [1865] (Apr. 1866).
Satsuma melleum (sic.), Lai 1990: 50.

####### Type locality.

Formosa [Taiwan] (Swinhoe)

####### Material examined.

*Lectotype*. Formosa, coll. Swinhoe, from collection of H. Cuming (NHMUK 20040576/1), new designation, 11 × 23.2 mm, whorls 5.

*Paralectotypes*. Formosa, coll. Swinhoe, from collection of H. Cuming (NHMUK 20040576/2–3, 2 shells).

####### Remarks.

The largest specimen mostly similar to the illustration in Pfeiffer (1866) is designated as the lectotype.

###### Satsuma sphaeroconus

Taxon classificationAnimaliaStylommatophoraCamaenidae

(Pfeiffer, 1866)

http://www.taibif.tw/en/catalogue_of_life/page/676b-ddd0-aa05-0ce7-2977-e3c7-8bb4-25e3-namecode-402928

Figure 4D

Bulimus sphaeroconus Pfeiffer, 1866: 830, pl. 46 fig. 3. [1865] (Apr. 1866).
Satsuma (Coniglobus) sphaeroconus , Kuroda and Habe 1949: 59.

####### Type locality.

Formosa [Taiwan] (Swinhoe)

####### Material examined.

*Lectotype*. Formosa, coll. Swinhoe, from collection of H. Cuming (NHMUK 20040575/1), new designation, 23.1 × 18.5 mm, whorls 6.

*Paralectotype*. Formosa, coll. Swinhoe, from collection of H. Cuming (NHMUK 20040575/2, 1 shell, immature).

####### Remarks.

The only mature shell with a red spot is designated as the lectotype.

###### Satsuma succincta

Taxon classificationAnimaliaStylommatophoraCamaenidae

(H. Adams, 1866)

http://www.taibif.tw/en/catalogue_of_life/page/4aa0-b629-2eb1-14c9-054a-7754-f168-a2e8-namecode-402929

Figure 4C

Helix (Camaena) succincta H. Adams, 1866: 316–317, pl. 33 figs. 4, 4a.
Satsuma succincta , Richardson 1985: 273.

####### Type locality.

Takow, Formosa [northern Qi-Jin, Kaohsiung City] (Swinhoe).

####### Material examined.

*Lectotype*. Takow Mountain, Formosa, coll. Swinhoe (NHMUK 1866.5.9.9/1), new designation, 22.8 × 31.2 mm, whorls 6.5.

*Paralectotypes*. Takow Mountain, Formosa, coll. Swinhoe (NHMUK 1866.5.9.9/2–6, 5 shells; NHMUK 1866.5.9.9/7, 1 shell, non *succincta*, immature shell of *Aegista lautsi branchylasis* (Schmacker & Boettger, 1891)).

####### Remarks.

One of the specimens is labelled "Takow mountains" on base of shell. It is here selected as the lectotype for the stabilization of the name (ICZN 1999: Art. 74).

#### Genus *Yakuchloritis* Habe, 1955

##### Yakuchloritis hungerfordianus

Taxon classificationAnimaliaStylommatophoraCamaenidae

(Moellendorff, 1884)

http://www.taibif.tw/en/catalogue_of_life/page/ac86-40bf-dd43-3659-1e45-9174-49a8-8ff4-namecode-316613

Helix hungerfordiana Moellendorff, 1884: 336–337, pl. 7 fig. 7.
Yakuchloritis hungerfordianus , Chang 1990: 36.

###### Type locality.

insulae Hongkong [Hongkong Islands] (Moellendorff)

###### Material examined.

*Lectotype*. Hong Kong (SMF 45429), designated by Zilch (1966b, fig. 42).

*Paralectotypes*. Hong Kong and Guangdung, China (SMF, 11 shells) (Zilch 1966b).

*Possible paralectotypes*. Keelung, Formosa, coll. Hungerford (NHMUK 1891.3.17.5–6, 2 shells), 9.6 × 18.1 mm, whorls 5.5; 9.7 × 16.5, whorls 5.5.

###### Remarks.

This species was named by Nevill, in a letter to Hungerford, on the basis of the samples that Hungerford collected from Formosa; however, the species was not published until 1884 by Moellendorff. Having examined Moellendorff’s collection from Hong Kong and Guangdung (Moellendorff 1884), I cannot find evidence of whether the lot in the NHM was actually seen by Moellendorff. Since he listed Formosa as one of the localities, I consider the specimens in the NHM to be possible paralectotypes.

#### Genus *Moellendorffia* Ancey, 1887

##### *Moellendorffia hiraseana* Pilsbry, 1905

###### Syn. Stegodera (Trihelix) helleri

Taxon classificationAnimaliaStylommatophoraCamaenidae

Rolle, 1911

http://www.taibif.tw/en/catalogue_of_life/page/1c56-7520-15b7-106a-322e-2f3c-77bb-032c-namecode-316609

Moellendorffia (Trihelix) hiraseana Pilsbry, 1905: 66–67, pl. 2 fig. 4–6.
Stegodera (Trihelix) helleri Rolle, 1911: 31–32.

####### Type locality.

Toyenmongai auf Formosa [Dong-yuan-men-jie, Tainan = central area of modern-day Tainan City] (Rolle?)

####### Material examined.

*Lectotype* of *Stegodera helleri*. Toyenmongai, Formosa, coll. Rolle, 1910 (SMF 7404), designated as holotype by Zilch (1966a, fig. 57), see remarks below.

*Paralectotypes* of *Stegodera helleri*. (SMF, 4 shells) (Zilch 1966a).

*Possible paralectotypes* of *Stegodera helleri*. Toyenmongai, Formosa, coll. Rolle, “1/2/11”, from collection of V.W. MacAndrew, no. 1563 (NHMUK 20040594, 4 shells), 6.4–6.9 × 15.9–17.4mm, whorls 4.75.

####### Remarks.

No holotype was originally fixed by Rolle (1911). Zilch (1966a) considered a specimen in SMF as the holotype, which here is accepted as a subsequent lectotype designation, and thus all other specimens from Rolle’s original lot receive the status of paralectotypes under Art. 74.6, ICZN (1999).

## Supplementary Material

XML Treatment for Cyclotus taivanus

XML Treatment for Platyrhaphe minutus

XML Treatment for Platyrhaphe swinhoei

XML Treatment for Ptychopoma wilsoni

XML Treatment for Chamalycaeus hungerfordianus

XML Treatment for Dioryx swinhoei

XML Treatment for Diplommatina hungerfordiana

XML Treatment for Pupinella swinhoei

XML Treatment for Pupinopsis morrisonia

XML Treatment for Pupina adamsi

XML Treatment for Euphaedusa sheridani

XML Treatment for Hemiphaedusa exilis

XML Treatment for Hemiphaedusa similaris

XML Treatment for Oospira formosensis

XML Treatment for Oospira swinhoei

XML Treatment for Elma swinhoei

XML Treatment for Videnoida shermani

XML Treatment for Petalochlamys hypograpta

XML Treatment for Petalochlamys vesta

XML Treatment for Lamprocystis fulgida

XML Treatment for Aegista fulvicans

XML Treatment for Aegista granti

XML Treatment for Pseudobuliminus incertus

XML Treatment for Acusta assimilis

XML Treatment for Acusta toyenmongaiensis

XML Treatment for Nesiohelix swinhoei

XML Treatment for Dolicheulota formosensis

XML Treatment for Dolicheulota swinhoei

XML Treatment for Satsuma bacca

XML Treatment for Eulota (Euhadra) warburgi

XML Treatment for Satsuma bairdi

XML Treatment for Satsuma formosensis

XML Treatment for Satsuma mellea

XML Treatment for Satsuma sphaeroconus

XML Treatment for Satsuma succincta

XML Treatment for Yakuchloritis hungerfordianus

XML Treatment for Stegodera (Trihelix) helleri
